# Description of the Cattle and Small Ruminants Trade Network in Senegal and Implication for the Surveillance of Animal Diseases

**DOI:** 10.1155/2023/1880493

**Published:** 2023-10-12

**Authors:** Mamadou Ciss, Alessandra Giacomini, Mame Nahé Diouf, Alexis Delabouglise, Asma Mesdour, Katherin Garcia Garcia, Facundo Muñoz, Eric Cardinale, Mbargou Lo, Adji Marème Gaye, Mathioro Fall, Khady Ndiaye, Assane Gueye Fall, Catherine Cetre-Sossah, Andrea Apolloni

**Affiliations:** ^1^Laboratoire National de l'Elevage et de Recherches Vétérinaires (ISRA-LNERV), Institut Sénégalais de Recherches Agricoles, Dakar-Hann BP 2057, Senegal; ^2^CIRAD, UMR ASTRE, Montpellier, France; ^3^CIRAD, INRAE, UMR ASTRE, Univ Montpellier, France; ^4^Department of Biosciences, Swansea University, Swansea SA2 8PP, UK; ^5^Direction des Services Vétérinaires, Dakar, Senegal

## Abstract

Livestock mobility, particularly that of small and large ruminants, is one of the main pillars of production and trade in West Africa: livestock is moved around in search of better grazing or sold in markets for domestic consumption and for festival-related activities. These movements cover several thousand kilometers and have the capability of connecting the whole West African region, thus facilitating the diffusion of many animal and zoonotic diseases. Several factors shape mobility patterns even in normal years and surveillance systems need to account for such changes. In this paper, we present an approach based on temporal network theory to identify possible sentinel locations, i.e., locations where pathogens circulation can be detected in the early phase of the epidemic (before the peak), using two indicators: *vulnerability* (i.e., the probability of being reached by the disease) and *time of infection* (i.e., the time of first arrival of the disease). Using these indicators in our structural analysis of the changing network enabled us to identify a set of nodes that could be used in an early warning system. As a case study, we simulated the introduction of transboundary animal diseases in Senegal and used data taken from 2020 Sanitary certificates (*laissez-passer sanitaire* (LPS)) issued by the Senegalese Veterinary Services to reconstruct the national mobility network. Our analysis showed that a static approach can significantly overestimate the speed and the extent of disease propagation, whereas temporal analysis revealed that the *reachability* and *vulnerability* of the different administrative departments (used as nodes of the mobility network) change over the course of the year. For this reason, several sets of sentinel nodes were identified in different periods of the year, underlining the role of temporality in shaping patterns of disease diffusion.

## 1. Introduction

The West African region includes the southern part of the bulge in the African continent and is crossed by the Sahel, a transitional strip between the Sahara Desert in the north and the Sudanic zone in the south [1]. The region is composed of 18 countries and is bounded in the north by Mauritania, Mali, and Niger, in the east by Chad and Cameroon, and in the south and west by the Atlantic Ocean. The region is characterized by different climates, and hence, different agroecological zones and different livestock farming systems [2]. Livestock farming (particularly cattle and small ruminants) is one of the most important economic activities in this area.

In West Africa, livestock mobility is an intrinsic component of livestock production and trade. The harsh environmental conditions, as well as the absence of the facilities required to slaughter animals and store meat, means livestock has to be mobile. To optimize the use of natural resources such as pasture and surface water, whose availability varies throughout the year, livestock farmers are forced to move their herds around: these movements occur all the year round (nomadism) or in specific periods (transhumance). Because of the lack of storage facilities and infrastructure, the majority of animals are sold alive at markets all year round. Most animals are concentrated in the northern part of West Africa, notably in Mali, Chad, Niger, and Mauritania, where the vast uninhabited areas are unsuitable for agricultural farming but allow extensive livestock raising, and the animals are moved toward the greener southern coastal areas. These movements are seasonal and depend both on the availability of resources and on other sociocultural factors, and the mobility patterns and the distribution of the volume of animals involved change over the course of the year [3, 4]. These movements integrate the region, connect contrasted agroecological areas, and, in addition, generate income for many supply chain actors, including producers, traders, transporters, and vendors, and contribute to the food and nutrition security of the region [5].

As it is, mobility in West Africa is a complex phenomenon involving different temporal scales (from a few days to several months) and spatial scales (from a few kilometers to reach local markets to international transhumance and/or international trade), and whose determinants range from environmental factors, e.g., the availability of natural resources, commercial factors, e.g., market demand and prices, to social factors, such as religious festivals [3].

In Senegal, livestock production is one of the main economic activities; it involves 28% of the population [6] and provides almost 4% [7] of gross national domestic product. Due to the different agroecological zones, several production systems coexist. Senegal is located on the Atlantic Coast axes of the transhumance routes (from Mauritania and Mali to Guinea and Guinea-Bissau) and is involved in international trade movements. Within Senegal, trade follows a strict market hierarchy: from village markets to consumption markets in coastal areas. National transhumance involves movements from the central and southern predominantly agricultural area toward the area of Ferlo in the north.

Like in other West African countries, movements within and toward Senegal vary over the course of the year. This is particularly true of the Tabaski religious festival, an important Muslim festival characterized by the sacrifice of rams, and the Grand Magal of Touba, during which the consumption of beef increases significantly. The two festivals mean imports of livestock increase enormously in a short period of time [3, 8]. Nevertheless, external events like the COVID-19 pandemic could strongly affect the flow of animals. In 2020, during the COVID-19 pandemic, several restrictive measures were introduced in Senegal that affected both human and livestock mobility. Borders were closed for both humans and animals in March 2020 and, at the same time, movements between regions were regulated. To supply markets and families in preparation for the Tabaski festival on July 31, borders were reopened 45 days before Tabaski and measures were eased for national and international movement (lettre circulaire n° 01806 PR/MESG/CT-PSS du 17 juin 2020). Similar decisions were taken on the occasion of the Grand Magal of Touba, a religious pilgrimage during which a large number of cattle in particular are sold and consumed. The application of these restrictions had a huge effect on the structure of the networks and on the risk of introduction and diffusion of pathogens, as reopening the border. It is important to note that normally, there are other religious festivals, like Gamou of Tivaouane, in addition to the Grand Magal of Touba, but these were canceled due to COVID-19 pandemic.

Animal movements also mean pathogens can be introduced and spread at national and international scales. Such pathogens spread very rapidly across national borders and have serious socioeconomic and public health consequences. Some of these, such as contagious bovine pleuropneumonia (CBPP), foot-and-mouth disease (FMD), peste des petits ruminants (PPR), and Rift Valley fever (RVF), are currently a major problem in West Africa [3, 4, 9, 10].

The porosity of the border, the absence of an animal identification system, together with the lack of coordinated control and surveillance systems hinders the development of a regional surveillance system and increases the risk of epidemics [3]. Understanding mobility patterns, as well as their variations, is of the uttermost importance to optimize surveillance and control systems. Senegal is one of the few countries in West Africa already equipped with a system for mapping and controlling animal movements within its borders. Movements are regulated through the use of sanitary certificates (*laissez-passer sanitaire* (LPS)), issued by the veterinary services to livestock transporters every time they move animals. The certificates are also routinely collected and centralized by the veterinary services. The information that can be retrieved from these data provides a snapshot of the livestock mobility network at each period of the year and could be used to develop tools to improve the surveillance system, adapted to the period concerned.

Network-based approaches are widely used in veterinary epidemiology to study the role of animal mobility in the spread of diseases, with the aim of developing effective strategies for disease surveillance and control [11, 12]. Network-based approaches make it possible to depict livestock movements as a spatial network in which the nodes represent villages, administrative units, markets, or herds, and a link is created each time at least one animal is moved from one node to another. However, while network methods have been extensively applied to engineer surveillance system in European countries, thanks to the existence of vast live animal movement traceability datasets (i.e., Lentz et al. [13] and Schirdewahn et al. [14]), little has been done in West Africa due to the scarcity of such information [15]. Only a few articles that report network analysis in West Africa have been published recently, including Apolloni et al. [16] and Nicolas et al. [17] for Mauritania, Jahel et al. [18] for Senegal and Mauritania, and Valerio et al. [19] for the whole West Africa region.

Static network approaches may not be the best way to create effective surveillance and control tools against the spread of infectious diseases, as a static approach can overestimate or underestimate the rate and extent of outbreaks [20]. However, many real networks are dynamical and change their structure over time (*temporality*): at each time, new nodes can appear and create new links; nodes already present can disappear or create/destroy links with other nodes. The influence of temporality on the structure of the network can significantly affect the spread of a disease, which consequently can only be accurately predicted if the chronology of links is accurately represented [20, 21].

In this work, we used a temporal network approach to assess the influence of structural changes (i.e., creation and disappearance of nodes and links) on the diffusion of animal diseases over time. We used data collected in 2020 by the Senegalese Veterinary Services to build a representation of the network, and adapted tools from complex networks to assess the risk of being infected and the role of different Senegalese areas in spreading infections over the course of the year. To this end, we relied on measures of the *vulnerability* and *reachability* of nodes. *Vulnerability* gives an indication of the likelihood a node will be infected, while *reachability* gives an indication of the time to infection. This approach takes changes in the network over time into account, as well as the network structure, and differs markedly from the static, individual-centric approaches used in previous risk assessments [22–25]. The objective of the present work is to provide a theoretical basis for improving the Senegalese surveillance system by identifying different potential geographical spots that contribute to the spread of pathogens at different times of the year and that could be used as sentinel nodes.

## 2. Materials

### 2.1. Study Area

Bordering Mauritania to the north, Mali to the east, Guinea and Guinea-Bissau to the south, and the Gambia and the Atlantic Ocean to the west, Senegal occupies an area of 196,722 km^2^ and, in 2020, had an estimated population of more than 16.7 million [26].

The administration of the Senegalese territory is organized in 14 regions, 45 departments, and 121 *arrondissements* [27]. There is a clear contrast between the empty area in the east (hosting around 10% of the human population of Senegal) and the populated and urbanized central areas in the west, where 90% of human population is concentrated, of which 25% in the Dakar area [7, 26].

Senegal climate is very varied and distinct climatic zones are characterized by different levels of rainfall and different types of vegetation. This diverse climate strongly influences the livestock farming sector, whose different farming systems depending on agroclimatic gradients, among other factors [8].

As mentioned above, the livestock trade is organized in a strict hierarchical system starting at village weekly markets (*Lumo*); the animals are collected by traders to be sold at collection markets before being sent on to consumer markets, where they are sold to be slaughtered. Because there are practically no meat storage facilities, most trade involves live animals. Livestock trade routes converge on the Dakar region, the main consumer market, with stops in smaller markets such as Saint-Louis, Touba, Thiès, and Kaolack. Before reaching the urban markets, the vast majority of the animals originating from northern Senegal, Mauritania, and Mali are grouped in Dahra, called the “livestock capital” of Senegal. Another collection market in the southeastern part of the country also plays a major role in the livestock trade: Tambacounda, the point of convergence for animals from eastern and southern Senegal, as well as from southern Mauritania and Mali. In addition to the movement of animals for sale, transhumance is widespread in Senegal, both at national scale from the central area to the north (in particular the Ferlo region), and, due to its location, international, from Mali and Mauritania to the Senegalese coast [3, 8] (Figure S1).

### 2.2. Data

In Senegal, a certification system based on a sanitary pass (LPS, from the French “Laissez-Passer Sanitaire”) is used to track animal mobility and to map the most important axes of movement in the region. Veterinary posts belonging to the Senegalese Ministry of Livestock and Animal Production provide an LPS each time a herd is moved; the document states the origin of the movement (village, department, region, country), the destination (village, department, region, country), the date, the species and number of animals involved, and the means of transport (Table S1). Copies of the LPS are centralized and stored in electronic form.

We focused our analyses on movements of cattle and small ruminants (goats and sheep), either separately or together. For analytical purposes, the two were aggregated on the spatial scale of an administrative department (all 45 Senegalese departments are involved in this trade) and on a time scale of 1 month or 1 week, depending on the type of analysis: we chose a month as the temporal unit for the general description of the data and for cluster analysis, and a week to simulate the disease spread, as a week is a more realistic unit to study disease propagation.

## 3. Methods

### 3.1. Descriptive and Network Analyses

We analyzed mobility data using a complex network approach. LPS data were used to build three oriented and weighted networks, one for each species plus one species-independent network: the nodes corresponded to the departments of origin and destination; a direct link existed between two nodes if at least one animal was moved from the department of origin to the destination department; the link was weighted according to the number of animals moved along it.

A cluster analysis was performed to explore the behavior of the different nodes over the study period. Due to the large variations on the number of animals exchanged, nodes were ranked based on their activity, defined as the effective number of animals traded each month; the number being positive if the inflow of animals was greater than the outflow (*importing behavior*), otherwise negative (*exporting behavior*). Clusters were then identified based on the activity pattern (i.e., the vector of node's ranking of each month) of each node along the year. A similar approach was used by Csáji et al. [28] to identify different activity areas in Portuguese urban areas.

Clustering was performed using hierarchical clustering on principal components (HCPC), which successively applies three standard methods used in multivariate analyses: (i) principal component analysis (PCA), which identifies the principal components, (ii) hierarchical clustering, which defines the optimal number of clusters of nodes according to their score on the principal components, and (iii) nonhierarchical clustering (in particular the k-means algorithm), which associates a cluster with each node [29].

To study the structure of the livestock network, we conducted a spatiotemporal analysis of link's frequency, defined as the number of months in the year in which movements occur on the link. We considered a link to be active when at least one trade movement was recorded in a given month. We then categorized the links according to the number of months in which they were active. In particular, we identified four frequency categories, which were, starting from the least frequent: occasional (activity only occurred in 1 month per year), intermediate (activity occurred in 2 or 3 months per year), frequent (activity occurred in 4–9 months per year), and backbone (activity occurred in 10–12 months).

To compare the risk of diffusion over the course of the year, we used the epidemic threshold *q* [30]. This measure provides information on the minimum probability for a virus to spread throughout the network: the lower the value of the epidemic threshold, the higher the risk of propagation.

For a weighted network, this parameter can be estimated as follows:

(1) $$\begin{array}{c} q_{w}=\frac{\sum_{i}w_{i}^{\text{out}}}{\sum_{i}w_{i}^{\text{out}}w_{i}^{\text{in}}}, \end{array}$$

where the sum is extended over all the nodes in the network at a specific month indicates the average value, *w*_*i*_^in^ and *w*_*i*_^out^ indicate the *i*-node's in-strength and out-strength (in-strength and out-strength correspond to the number of incoming and outgoing animals to/from a specific node), respectively [17].

Following the procedures of Lancelot et al. [31] and Nicolas et al. [17], for each of the three mobility networks considered (all species, cattle, small ruminants separately), the epidemic threshold was estimated for each monthly snapshot of the network to assess the risk of an epidemic occurring over the course of the year.

### 3.2. Identification of Potential Sentinel Nodes

**Figure 1:**
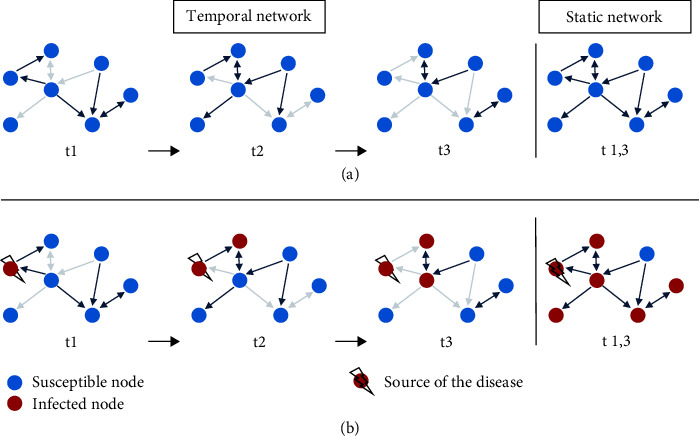
(a) An example of a directed temporal network and its static counterpart. The dark links are those in the temporal snapshot, while the pale links are those that are possible but are not present in the temporal snapshot. (b) Simple simulation of disease spread in the temporal network (on the left) and the static network (on the right).

Temporality, i.e., the variation in time of the mobility network, affects disease spread.  Figure 1(a) shows an example of a temporal network and its static counterpart. The network is composed of seven nodes and eight possible links, whose direction is indicated by the arrows. In this case, the temporal network is characterized by three temporal snapshots that contain the same nodes but different links. A link that is present and active in a snapshot is not necessarily the same in the previous or the following snapshots. If we disregard the information on timing, we obtain an aggregated/static network composed of the same nodes and links as the temporal network, all present and active at the same time. If we simulate an outbreak in the two networks (temporal and static) (Figure 1(b)), we can see that the potential diffusion of the pathogen differs in the two situations. In this case, there is significantly more propagation in the static network than in the temporal one. This happens because, in the temporal network, the disease can only propagate through temporal paths. In other words, if a link connecting an infected node to a susceptible one is active in a specific temporal snapshot, the disease can spread to the latter; conversely, if the link is not active in the temporal window concerned, disease propagation stops.

To study the influence of temporality on disease propagation, we used a deterministic susceptible-infected (SI) model similar to a message-passing one: the disease was transmitted from an infected node to a susceptible one with a probability of 1, and the infected nodes remained infected for the entire period of analysis, and were consequently able to continue to spread the disease even weeks after being infected. The aim of this procedure was to identify the set of nodes that could potentially be infected at each time when the underlying structure varied in time. This is a rough approximation but allows us to elicit the effect of structural changes (a node can get infected only if a link exists at specific times connecting to an infected one), identifying “potential” nodes at risk at each timestep, in a manner that is independent of particular disease under study (and its transmission probability), and avoiding the inherent stochasticity of the process. Results from this model correspond to the worst scenario case (all the nodes exposed to an infected node are automatically infected), but depend only on the structure of the network at specific time (i.e., the number of nodes in contact with infected nodes) and are disease independent.

Because of our focus on the control of transboundary animal diseases, the departments of Mali and Mauritania, which export livestock directly to Senegal, were chosen as sources of the disease, as the majority of Senegalese imports of small ruminants and cattle are from these two countries [3, 8].

To explore the effect of temporality on the structure of the network, and hence on diffusion of the disease, we compared results obtained with a static representation (in which the structure of the network remains unchanged throughout the year) with results obtained with a temporal representation. In the first case, all the links recorded in the dataset were present at the same time, the time of activation was not taken into account, while in the second case, we included changes in the structure in every week of the study period. To this end, we used temporal path formalism, according to which a temporal path is a sequence of links connecting two nodes with each link in the path coming temporally after the one before it [20]. This approach enabled us to estimate the *infection time*: that is the minimum number of timesteps (i.e., weeks) needed to create a temporal path between an infected node and the node under observation.

Among all the possible temporal paths between the sources and the other nodes, we decided to consider the “earliest arriving” paths, which represent the first time a node is infected by the disease [32, 33]. The speed/rate at which a node became infected was estimated by the *infection time*, i.e., the number of weeks that elapsed between the onset of the disease and the time at which the department concerned was reached for the first time. For static networks, the speed/rate of infection was estimated from the length of the shortest paths, converting the links into temporal units, specifically weeks. If a node was reached by more than one source, the shortest *infection time* (for temporal networks) or the shortest path (for static networks) was chosen.

All descriptive analyses and static/temporal network analyses were carried out using R software with the following packages: ggplot2 for graphs [34], ggplot2 and tmap for maps [35], FactoMineR [36] and factoextra [37] for cluster analysis, and sna [38] and tsna [32] for static and temporal network analyses, respectively. A freely available version for the deterministic SI model is available on gitlab using the command *git clone* git@forgemia.inra.fr:umr-astre/sentinel_nodes.git.

## 4. Results

### 4.1. Summary Statistics

The database contained information on 8,861 livestock trade movements from January to December 2020. The network is composed of a total of 88 nodes, corresponding to an administrative unit of level 2, of which 45 are Senegalese (departments) and 590 unique links, i.e., origin–destination combinations. The movements concerned Senegal as the origin and/or destination of 87% of the movements and eight other countries: Mali (9%), Gambia (2%), Mauritania (1%), Guinea, Guinea-Bissau, Burkina Faso, Niger, and Nigeria (<1% each) as either the origin or as the destination of movements. Focusing on Senegal, a total of 6,511 national movements and 2,350 international movements, over, respectively, 458 and 132 unique links, involving 87,017 cattle and 553,718 small ruminants, were recorded in the dataset. Despite the large number of national trades, the majority of animals were moved for the purpose of international trade. More than 95% of these movements were in trucks, which is the most widely used means of transport for animals in the region concerned. More than 600,000 animals were transported by truck, and the remainder mainly on foot (Table 1).

The livestock network was analyzed as static but also took temporality into account, which influences the presence/absence of links.

**Figure 2:**
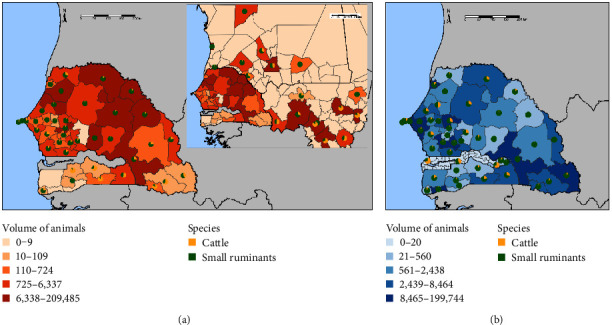
Distribution of the volume of animals in the administrative departments of Senegal, according to whether the department is the origin (a) or the destination (b) of livestock movement. The miniature pie charts show the percentage of cattle (yellow) and small ruminants (green) in the total number of animals. Quartiles were chosen for the colors representing the volume of animals traded. Only countries that account for at least 1% of exports (a) or imports (b) are shown.

As shown in Figure 2, all Senegalese administrative departments are involved in animal trade either as the origin, the destination, or both. Movements are both national and international, and, while Senegal is the final destination of almost all the trade, many animals are moved not only from other Senegalese departments, but also from Mali and Mauritania, the main exporters, with some departments, particularly in Mali, exporting a significant number of animals (Figure 2(a)).

The departments in northeastern Senegal (Podor, Matam, Kanel, and Ranérou Ferlo) are notable for their high level of animal “exports.” Other Senegalese departments (Tambacounda in the south, Koungheul and Gossas in the center, Louga and Kébémer in the north) also export considerable numbers of animals.

Concerning imports, the departments that import the most animals are located in the Dakar region, in particular Pikine, Rufisque, Thiès, and M'bour, where the majority of consumer markets are located. Other Senegalese departments that import large numbers of animals are Saint-Louis in the north, Mbacké and Guinguinéo in the center, Ziguinchor in the southwest, and Tambacounda and Sayara in the southeast, on the border with Mali (Figure 2(b)).

**Figure 3:**
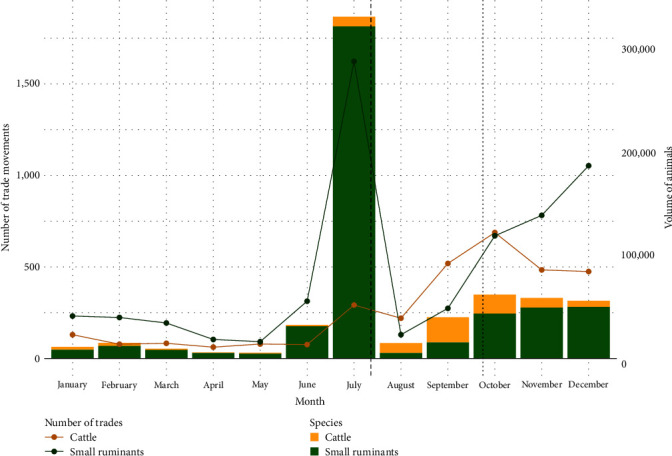
Number of trade movements (line plot) and volume of livestock traded (bar plot) recorded in 2020 per species and per month. Data concerning cattle are in yellow, and data concerning small ruminants are in green. The dashed line represents the Tabaski festival (July 31), and the dotted line represents the Grand Magal of Touba festival (October 6).

Figure 3 shows the number of movements and the volume of animals traded in each species (cattle or small ruminants) per month. Overall, movements of animals for the purpose of trade were less frequent in the first 6 months of the year, but increased in July, particularly trade in small ruminants. Similarly, July was the month with the most trade in small ruminants in the study period, involving more than 300,000 animals. In August and September, the volume of small ruminants decreased, while both the movement and volume of cattle traded increased, overtaking those of small ruminants. In October, November, and December, the number of cattle trades decreased, but remained higher than in the rest of the year, while the number and volume of trade in small ruminants increased, although less sharply. In 2020, two important Muslim festivals took place at the end of July (Tabaski) and at the beginning of October (Grand Magal of Touba) and are represented on the chart by a dashed line and a dotted line, respectively (Figure 3).

In the whole year, trades of small ruminants were concentrated on 503 links and trades in cattle on 329 links, including 242 links trades of both species. Like for small ruminants, the highest number of unique trade links occurred in July, followed by, in decreasing order, December, November, and October, also the months with the highest number of trade links for cattle. The links used by the two species also increased in the last 3 months of the year (Table S2).

Concerning the means of transport, trucks were used for almost all movements of animals for sale throughout the year. The number of movements peaked in July, and, then after a significant drop, started to increase again in September (Table S3).

### 4.2. Cluster Analysis

**Figure 4:**
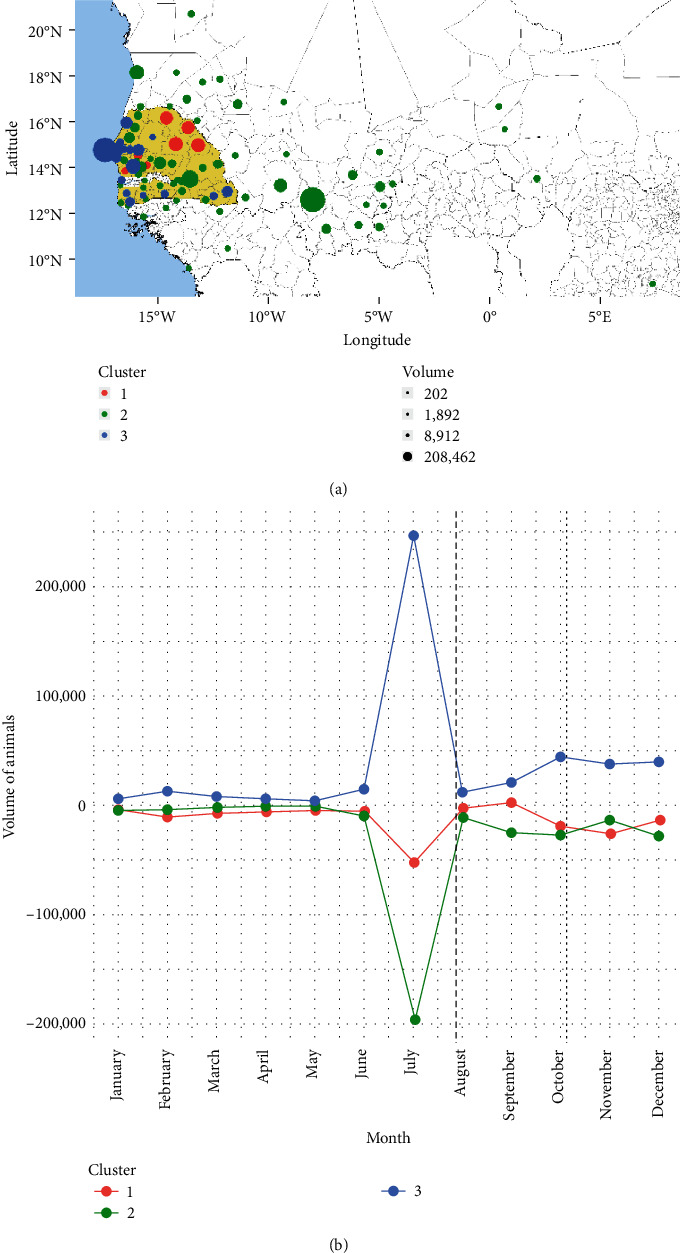
Clustering of livestock network. (a) Spatial representation of nodes colored according to the cluster to which they belong. The size of each dot indicates the volume of animals traded over the course of the year and the division is made in quantiles. (b) Temporal representation of trade by the three clusters over the course of the year, in terms of the volume of animals traded. Imported animals are represented as positive numbers, and exported animals are represented as negative numbers. The dashed line represents the Tabaski festival on July 31, and the dotted line represents the Grand Magal of Touba festival on October 6.

Figure 4 shows the nodes of the livestock network in three (3) clusters:

- Cluster 1, composed of seven nodes and characterized by a “weak” exporting behaviour.
- Cluster 2, composed of 61 nodes and characterized by a “strong” exporting behavior.
- Cluster 3, composed of 20 nodes and characterized by a “strong” importing behavior.

We introduce the terms *importing and exporting behavior* to indicate those nodes whose net flow of animals (difference between inflow and outflow) through them is positive and negative, respectively. Weak and strong refer to magnitude of the net flow (small or large).

Cluster 1 (in red) aggregates seven nodes, of which four are located on the northeastern border of Senegal (Matam, Podor, Kanel, and Ranérou Ferlo) with high volumes of animals traded, while the other three (Foundiougne, Kaffrine, Gossas) are located on the southern border of Dakar region (Figure 4(a)). However, in September, Cluster 1 imports are “weak,” with a slightly less than 3,000 animals imported (Figure 4(b)).

Cluster 2 (in green) aggregates all the foreign nodes, except Banjul (Gambia), and several nodes across Senegal, accounting for a total of 61 nodes out of 88. Cluster 2 is “strong” in terms of volume of animals exported over the year, despite the fact some nodes import more than export. Some nodes that export large numbers of livestock include Bamako (Mali, 208,462) and Nouakchott (Mauritania, 43,472), while Tambacounda (Senegal, 70,845) is a good example of an importing node (Figure 4(a)). The highest number of exports by this cluster occurred in July, when the number of animals exported was slightly under 200,000 (Figure 4(b)).

Cluster 3 (in blue) aggregates 20 nodes of which the majority is concentrated in the Dakar region but includes some nodes in southern Senegal and one foreign node, Banjul (Gambia). Of the nodes located in southern Senegal, two are on the border with Mali (Saraya and Kédougou), while the other four are located farther west. All the nodes in this cluster were characterized by strong import trade, with most imported animals via Pikine (Senegal, 199,703), but also via Thiès (Senegal, 51,939) and Kaolack (Senegal, 46,432) (Figure 4(a)). Reflecting the movements of livestock for export, this cluster shows a peak of imported animals in July, with a volume of around 250,000 animals, and another, less significant increase from September to October (Figure 4(b)).

### 4.3. Frequency of Links

**Figure 5:**
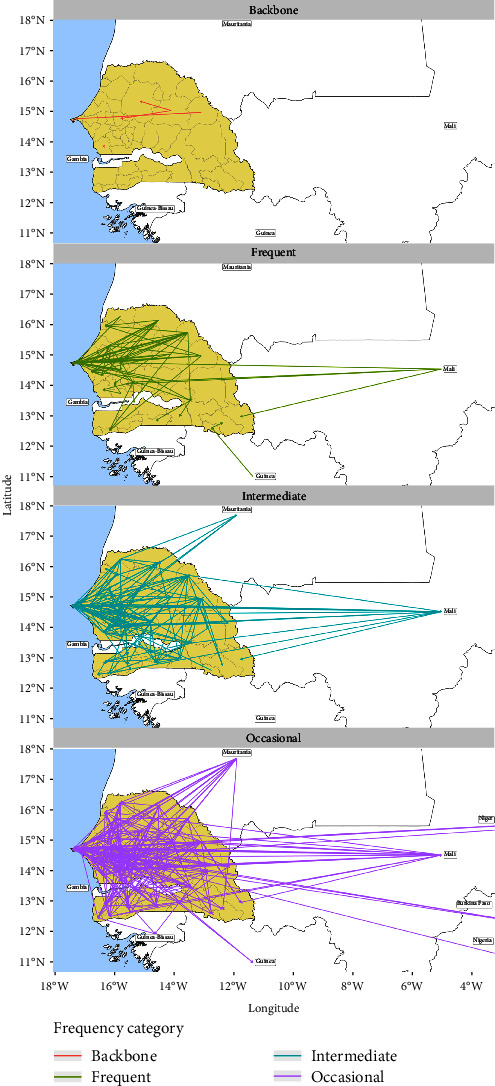
Geographical representation of the livestock network links by the frequency of their activity over the year. Backbone links were active in more than 9 months, frequent links were active between 4 and 9 months, intermediate links were active for 2 or 3 months, and occasional links were active for only 1 month of the study period. We decided to only show the administrative departments of Senegal on these maps. Therefore, concerning national trade, the origin and the destination are both Senegalese departments, while for international trades, they may be a Senegalese department or a central point in a foreign country.

Figure 5 shows the trade links divided by the frequency of their activity over the course of the year. In general, far more links were characterized by low and very low activity than by very high activity.

The backbone links are three national, short-range connections between northeastern and northwestern nodes: Kanel–Pikine, Ranérou Ferlo–Linguère, and Ranérou Ferlo–Mbacké. The majority of frequent links is concentrated in the north of Senegal, where several connections link eastern and western nodes, but some connections link northern and southern nodes. Moreover, some international links are frequent, in particular four originating from Mali and one from Guinea-Bissau. The number of intermediate links is significantly larger than that of the two previous categories, with several connections between Senegal and Mali, and between Senegal and Mauritania. Occasional links are extremely numerous and dense, with several connections in Senegal but also links to all its neighboring countries (Figure 5). The majority of intermediate and occasional links are active in July and October, due to the Tabaski and the Grand Magal of Touba religious festivals. However, considering the whole study period, frequent links are the most common (Figure S4).

### 4.4. Epidemic Threshold

**Figure 6:**
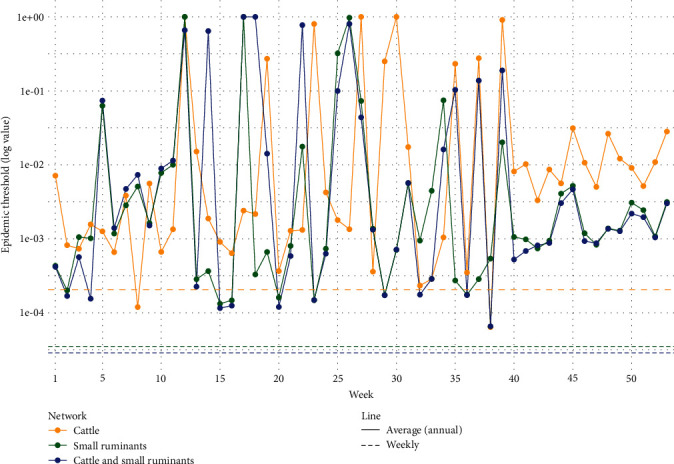
Logarithmic of the epidemic threshold over the course of the year in the three livestock mobility networks. Results for the small ruminants network are in yellow, the cattle network in green, and the combined livestock network in blue.

Overall, the values of the epidemic threshold of all three networks are extremely low, particularly for the combined livestock and the small ruminants network, whose results are almost identical. On the other hand, the epidemic threshold values of the cattle network (yellow curve in Figure 6) are higher overall, particularly toward the end of the year (Figure 6).

### 4.5. Identifications of Potential Sentinel Nodes

**Figure 7:**
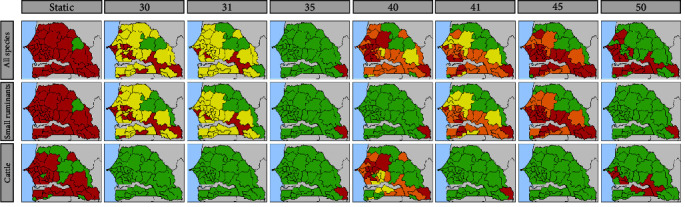
Geographical representation of infection time in the case of disease propagation from Mali. For each mobility network, the first column on the left represents the spread in the static network, and the other seven columns represent the seven worst scenarios of transmission if that specific week represents the beginning of the disease spread. The colors indicate the infection time of the disease: red for less than 1 month, orange for less than 2 months, yellow for more than 2 months, and green for nodes that have never been touched in 1 year time. For the static networks in the first column on the left, the colors are based on the number of links in the path: up to five in red, and green for nodes that have never been touched in 1 year time. Week 31 identifies the week of the Tabaski festival and week 41 identifies the week of the Grand Magal of Touba festival.

Maps focused on Senegalese departments were drawn to compare the results of the simulations run on the three networks in an efficient and easily understandable way (Figure 7). To assess the role of changes in the structure of the networks over time, and hence changes in disease spread, we compared the results of a static representation (in the column on the left) with those of a temporal representation (in the other seven columns). For the static representation, considering the time the outbreak began is meaningless, whereas for the temporal one, it is important, as the network structure can change over time. Therefore, each element in the seven columns representing the temporal networks corresponds to the results of an outbreak that began in a specific week of the year (the number of the week is given in the header of each map).

The departments are colored according to their *infection time*, i.e., the length of the period before they were reached by the virus. For each scenario, the *infection time* was estimated as the time (number of weeks) elapsed since the outbreak of the epidemic. We created four categories of *infection time*; each category is shown in a different color: red for departments infected less than 1 month from the beginning of the disease spread (less than 5 weeks), orange for departments infected after 1–2 months (between 5 and 9 weeks), yellow for departments infected after more than 2 months (more than 9 weeks), and green for those never reached by the disease. If a node was reached by infections from several sources, the shortest *infection time* was chosen.

For the static networks, we considered the number of links in the shortest path between the source and the node as weeks: red for the shortest paths with less than five links, orange for paths with between five and nine links, yellow for paths with more than nine links, and green for nodes that were never reached by the disease.

The results presented are those of the simulation of a disease spreading from Mali, the origin of most animals imported into Senegal in 2020. For the temporal networks, we present only a few weeks characterized by activity, in order to be able to simultaneously show changes over time and differences between the three networks. The complete results of the spread of a disease from Mali plus for a disease spreading from Mauritania can be found in Supplementary Information (Figures S5–S10).

Independently of the species involved and the network representation (temporal or static), the department of Kanel never gets infected because no movements have been recorded to this area in our dataset.

In general, in all three networks, maps representing aggregate networks strongly overestimated both the quantity of potentially infected nodes and the earliness of infection compared to those of temporal networks. In addition, there is a difference in the potential sanitary risk between the cattle temporal network and small ruminants temporal network, and the latter showing an average wider and potentially greater disease propagation. However, the combined network of cattle and small ruminants (the livestock network) is under the greatest sanitary risk.

Figure 7 also shows that, particularly for the livestock network and the small ruminants network, the periods around religious festivals (weeks 30 and 31 for the Tabaski, and weeks 40 and 41 for the Grand Magal of Touba) are characterized by a large number of infected departments, some of which are infected early.

## 5. Discussion and Conclusion

In our study, we considered the diffusion of a generic direct animal disease transmission and estimated the *vulnerability* and the *reachability* of nodes when the underlined network changes over time. In this way, we were able to identify Senegalese departments that could potentially be infected at the earliest stage of an epidemic. With a few modifications, our approach could be extended to include vector-borne diseases.

The structure of the Senegalese livestock network varies widely over the course of the year due to the seasonality of transhumance and the effect of religious festivals [3, 18] but, in 2020, these effects were exacerbated by the restrictive measures introduced as a result of COVID-19. In fact, around June and July, we noted a pickup in the movement and exchange of animals (mainly small ruminants) mainly due to the easing of the restrictive measures in preparation for the Tabaski festival and (mainly cattle) for the Grand Magal of Touba festival. We also noted that the dynamics of the small ruminants trade strongly drive the dynamics of the network as a whole.

Dakar is the main consumption area of Senegal because almost a quarter of the population of the country live in the city. Consequently, the main markets of Dakar and Pikine (at the entrance of Dakar) are the main destination of livestock movements. In particular, regular movements occur between the areas of Kanel, Ranerou Ferlo, Dahra, and the Senegalese capital. In these areas, there is a high concentration of collection markets (local name *lumo*), where traders frequently buy animals to be sold directly to Dakar or to the other collection markets in Dahra or Thiès before being sent on to the capital city. Overall, the majority of northern links end in the Dakar region or in some smaller but nevertheless important markets such as Saint-Louis, Thiès, Mbour, but also Ziguinchor in the south. Some links in the southeast start from Tambacounda, an important point of convergence for animals from eastern Senegal, as well as from Mauritania and Mali. Our analysis revealed that the role played by the different departments changes over the course of the year. Locations that are idle for a large part of the year become active during the Tabaski period and continue to be active until the end of the year, in particular, departments that produce small ruminants. Occasional and intermediate links that are active a few times a year are usually located near festival centers to support the increased supply of livestock, thereby increasing the sanitary risk.

Analysis of the threshold parameters showed that the network is prone to disease spread, but that the risk fluctuates over the course of the year. The risk increases significantly on the occasion of festivals due to the introduction of large numbers of animals and the creation of new commercial routes, and diseases can then spread easily across the network. However, the potential infected areas and the reachable time do not remain stable over the course of the year and this information cannot be captured using a static representation of the network. In fact, a static representation of the mobility patterns may largely overestimate the speed and the extent of disease diffusion: when the simple static approach is used, diseases appear to spread throughout the country in less than a month, whereas temporal analysis shows that *reachability* and *vulnerability* of departments vary over the course of the year. In most cases and depending on the species involved, few departments are reached in a month, although during the months around Tabaski and Grand Magal of Touba, the number of departments that can be reached increases drastically, and for some departments (like Dakar, Thiès, Tambacounda, and Dahra) where the main markets are located, and at the border, this risk is even higher. These results could be of great interest not only for risk-based surveillance but also for optimizing the distribution of resources and personnel needed for control at specific times of the year by focusing on the areas that are most likely to be reached. The fact that departments located at the border are most prone to early infection, means that sanitary control at the border should be strengthened and surveillance and control measures should be harmonized at regional level.

Previous works already underlined the importance of mobility and of data collection as a tool to improve surveillance and control in Africa [9, 12, 17]. Our work fits into this strand, emphasizing the importance of collecting data on animal mobility on a regular basis in order to retrieve information on structural changes. The objective of the present study is to provide theoretical tools to assess the importance of network dynamics when planning control and surveillance policies, using a limited set of information (i.e., only the origin and destination of movements at each time of the year) and avoiding numerical simulations. The methodology is generic enough to be applied to any disease. A more detailed analysis focused on specific diseases and that accounts for volume distribution could help assessing the probability for each node to be infected and help prioritizing the list of departments to monitor. To this end, further simulations are needed and their results will depend to a large extent on the characteristics of the disease concerned, e.g., it transmissibility and incubation period that could shape the spatiotemporal pattern of the epidemics and, hence, the involvement of the different departments. Future works should, thus, also consider stochastic models like Kim et al. [39] for specific diseases. In the model we used here, we aggregated data at the spatial scale of an administrative department, based on the assumption that the diffusion within a department is homogeneous. In practice, the presence of markets or transhumance corridors could attract movements in specific parts of the department, thereby increasing the risk in certain locations over the risk in other parts of the same department. Data on movements within departments were rare in our dataset (because of the way data were collected) and further field studies are recommended to collect data at a finer scale.

## Figures and Tables

**Table 1:** Summary of the characteristics of the data analyzed in the study.

	Trade movements	Headcount	Number of unique links
Species			
Cattle	3,186	87,017	328
Small ruminants	5,675	553,718	502
Type			
International	2,350	365,903	132
National	6,511	274,832	458
Means of transport			
Train	4	170	2
Truck	8,239	608,816	552
On foot	587	30,354	85
Boat	31	1,395	9

The number of movements, the number of animals, and the number of unique links are given for each species, type of trade, and means of transport, respectively.

## Data Availability

The livestock mobility data used to support the findings of this study are available from the DSV Senegal upon request.

## Supplementary Materials

Supplementary Materials Figure S1: map of Senegal colored based on the aridity index. Figure S2: volume of livestock traded in 2020 divided by week. Figure S3: graphical visualization of methods chosen to assess the number of clusters. Figure S4: activity of the links over the course of the year. Figure S5: geographical representation of infection time in the case of a disease propagated from Mali through the livestock network. Figure S6: geographical representation of infection time in the case of a disease propagated from Mali through the small ruminant network. Figure S7: geographical representation of infection time in the case of a disease propagated from Mali through the cattle network. Figure S8: geographical representation of infection time in the case of a disease propagated from Mauritania through the livestock network. Figure S9: geographical representation of infection time in the case of a disease propagated from Mauritania through the small ruminant network. Figure S10: geographical representation of infection time in the case of a disease propagated from Mauritania through the cattle network. Table S1: metadata of the dataset used in the article. Table S2: number of unique trade links in the small ruminant network and in the cattle network. Table S3: number of unique trade links according to the means of transport.
