# Supplementary material for: Description of the Cattle and Small Ruminants Trade Network in Senegal and Implication for the Surveillance of Animal Diseases
Source: Transbound Emerg Dis. 2023 Oct 12;2023:1880493. doi: 10.1155/2023/1880493 (PMC12016965; doi:10.1155/2023/1880493)
Supplement: Supplementary Materials — Figure S1: map of Senegal colored based on the aridity index. Figure S2: volume of livestock traded in 2020 divided by week. Figure S3: graphical visualization of methods chosen to assess the number of clusters. Figure S4: activity of the links over the course of the year. Figure S5: geographical representation of infection time in the case of a disease propagated from Mali through the livestock network. Figure S6: geographical representation of infection time in the case of a disease propagated from Mali through the small ruminant network. Figure S7: geographical representation of infection time in the case of a disease propagated from Mali through the cattle network. Figure S8: geographical representation of infection time in the case of a disease propagated from Mauritania through the livestock network. Figure S9: geographical representation of infection time in the case of a disease propagated from Mauritania through the small ruminant network. Figure S10: geographical representation of infection time in the case of a disease propagated from Mauritania through the cattle network. Table S1: metadata of the dataset used in the article. Table S2: number of unique trade links in the small ruminant network and in the cattle network. Table S3: number of unique trade links according to the means of transport. [file 1880493.f1.docx]

# Supplementary materials

| 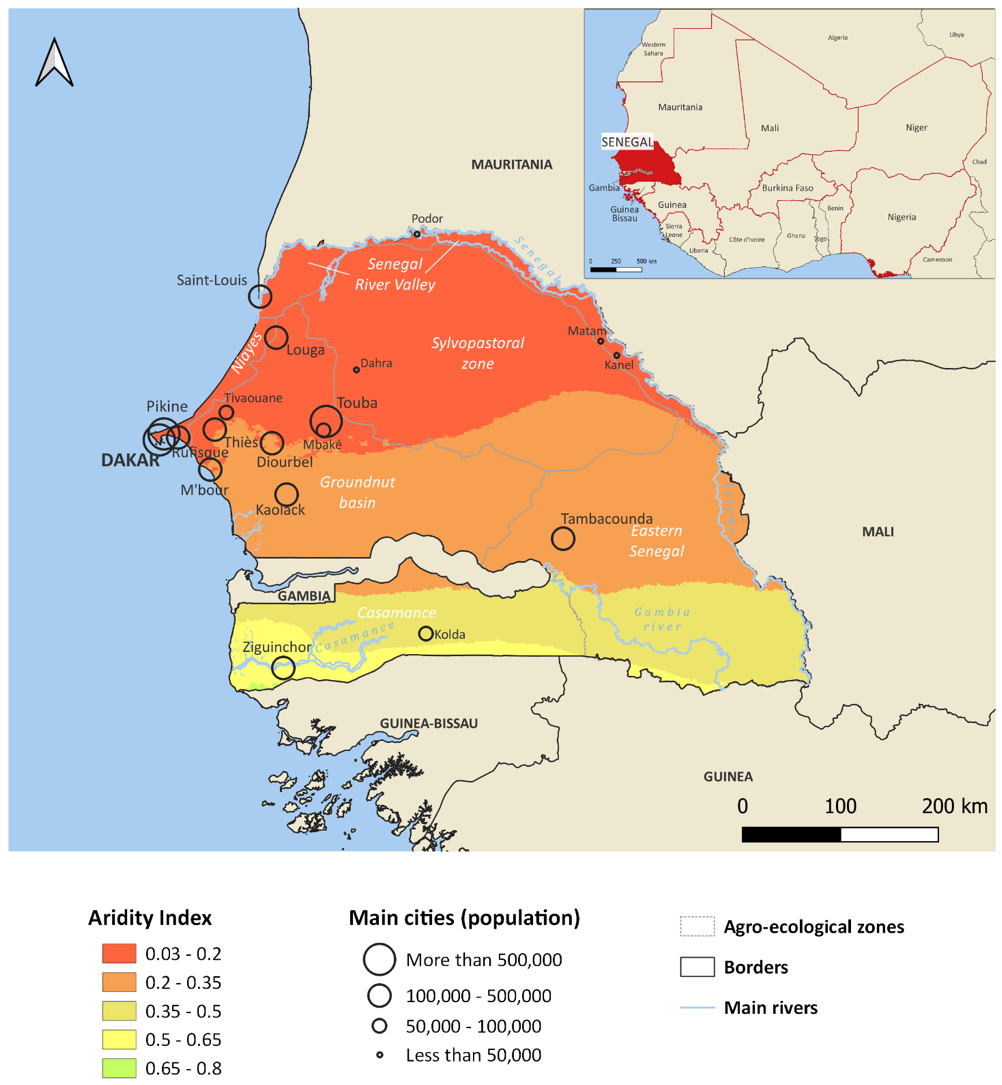 |
| --- |

Figure S1: Map of Senegal colored based on the aridity index. The inset map shows all the countries involved in livestock mobility network.

Table S1: Metadata of the dataset used in the article.

| Column name | Description | Type |
| --- | --- | --- |
| Origin | Village from which the livestock movement starts | character |
| Destination | Village at which the livestock movement ends | character |
| Number | Quantity of animals moved | integer |
| Species | Livestock type: small ruminants, cattle | character |
| Mean_transport | Mean of transport used to move animals: train, truck, walking, water | character |
| Date | Date of the livestock movement | date |
| Month | Month of the livestock movement: 1:12 | integer |
| Week | Week of the livestock movement: 1:53 | integer |
| Dept_or | Name of the origin department | character |
| Region_or | Name of the origin region | character |
| Country_or | Name of the origin country | character |
| Dept_dest | Name of the destination department | character |
| Region_dest | Name of the destination region | character |
| Country_dest | Name of the destination country | character |

Table S2: Number of unique trade links in the small ruminant network and in the cattle network. The values are represented divided by month, while the “whole year” line corresponds to the number of unique links considering the network as static. The last column shows the number of links that are used by the two species.

|  |  | Small Ruminants | Cattle | Links in common |
| --- | --- | --- | --- | --- |
| Whole year |  | 503 | 329 | 242 |
| Months |  |  |  |  |
|  | January | 42 | 27 | 13 |
|  | February | 52 | 27 | 22 |
|  | March | 59 | 30 | 19 |
|  | April | 26 | 18 | 12 |
|  | May | 27 | 22 | 11 |
|  | June | 71 | 28 | 20 |
|  | July | 202 | 47 | 37 |
|  | August | 29 | 21 | 6 |
|  | September | 24 | 41 | 14 |
|  | October | 131 | 123 | 66 |
|  | November | 163 | 113 | 69 |
|  | December | 184 | 128 | 82 |

Table S3: Number of unique trade links according to the means of transport. The values are given per month, while the “whole year” line corresponds to the number of unique links considering the network as static. The last column shows the number of links that are shared by the movements made by truck and those made by all other types of transport, including on foot.

|  |  | Truck | Others | | | Links in common |
| --- | --- | --- | --- | --- | --- | --- |
|  |  |  | Water | Walking | Train |  |
| Whole year |  | 552 | 9 | 85 | 2 | 55 |
| Months |  |  |  |  |  |  |
|  | January | 49 | 0 | 8 | 0 | 1 |
|  | February | 56 | 0 | 3 | 0 | 2 |
|  | March | 67 | 0 | 3 | 0 | 0 |
|  | April | 31 | 0 | 1 | 0 | 0 |
|  | May | 38 | 0 | 0 | 0 | 0 |
|  | June | 73 | 0 | 9 | 0 | 3 |
|  | July | 207 | 0 | 11 | 0 | 6 |
|  | August | 43 | 0 | 3 | 0 | 2 |
|  | September | 42 | 3 | 11 | 0 | 5 |
|  | October | 178 | 1 | 17 | 1 | 9 |
|  | November | 178 | 3 | 33 | 0 | 7 |
|  | December | 219 | 3 | 20 | 1 | 11 |

| 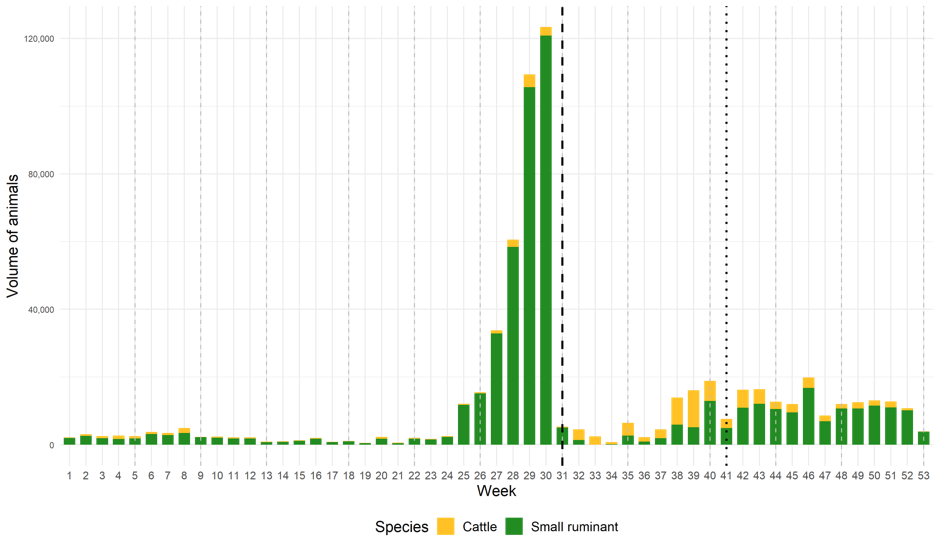 |
| --- |

Figure S2: Volume of livestock traded in 2020 divided by week. Cattle are shown in yellow and small ruminants are in green. The black dashed line represents the day of the Tabaski festival (July 31), the black dotted line represents the day of the Grand Magal of Touba (October 6). The months are indicated by the gray dashed lines.

| 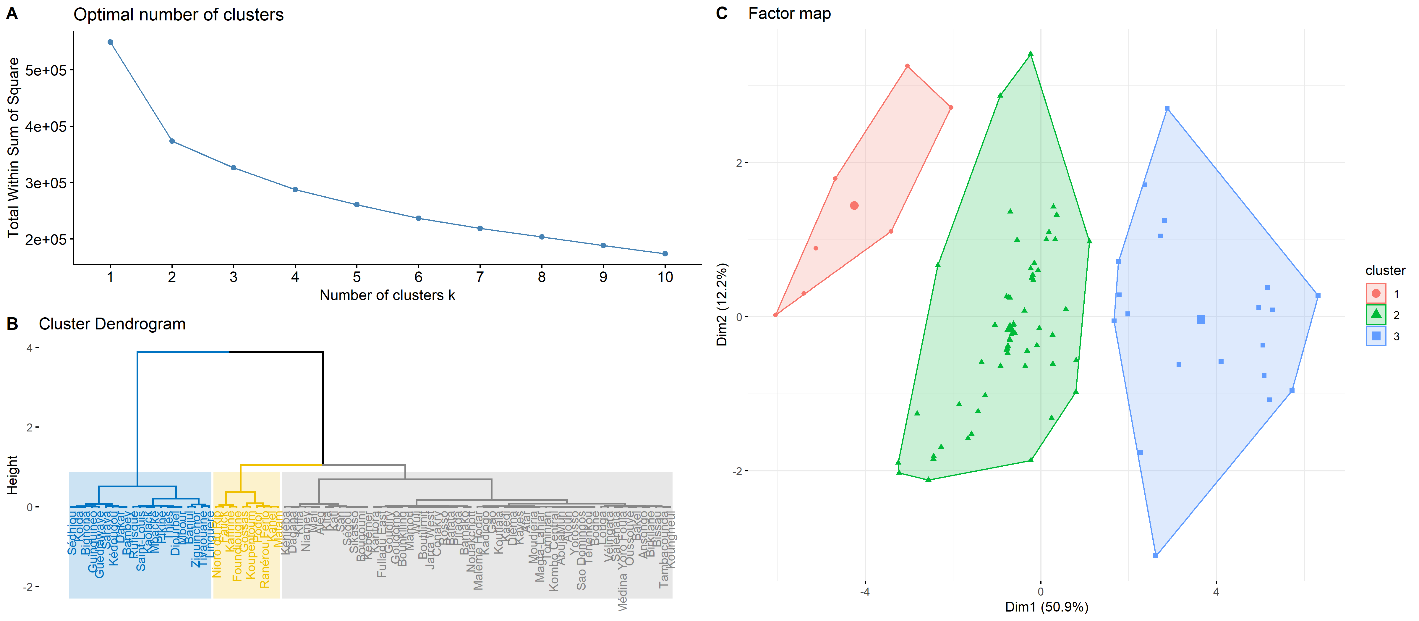 |
| --- |

Figure S3: Graphical visualization of methods chosen to assess the number of clusters: (A) Elbow method, (B) cluster dendrogram, and (C) cluster division with the HCPC (Hierarchical Clustering on Principal Components) function of the FactoMineR package.

| 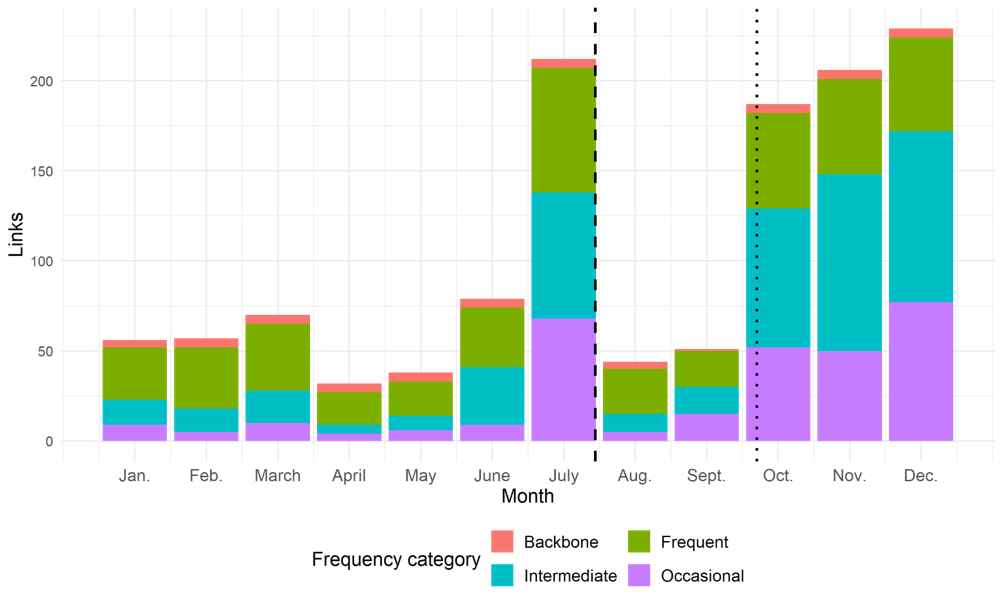 |
| --- |

Figure S4: Activity of the links over the course of the year. For each month, the quantity of active links is shown, colored according to their frequency. Backbone links are those active more than nine months a year, frequent links are those active from four to nine months, intermediate links are those active two or three months, and occasional links are only active one month. The orange line represents the Tabaski festival (July 31), the violet line represents the Grand Magal of Touba festival (October 6).

| 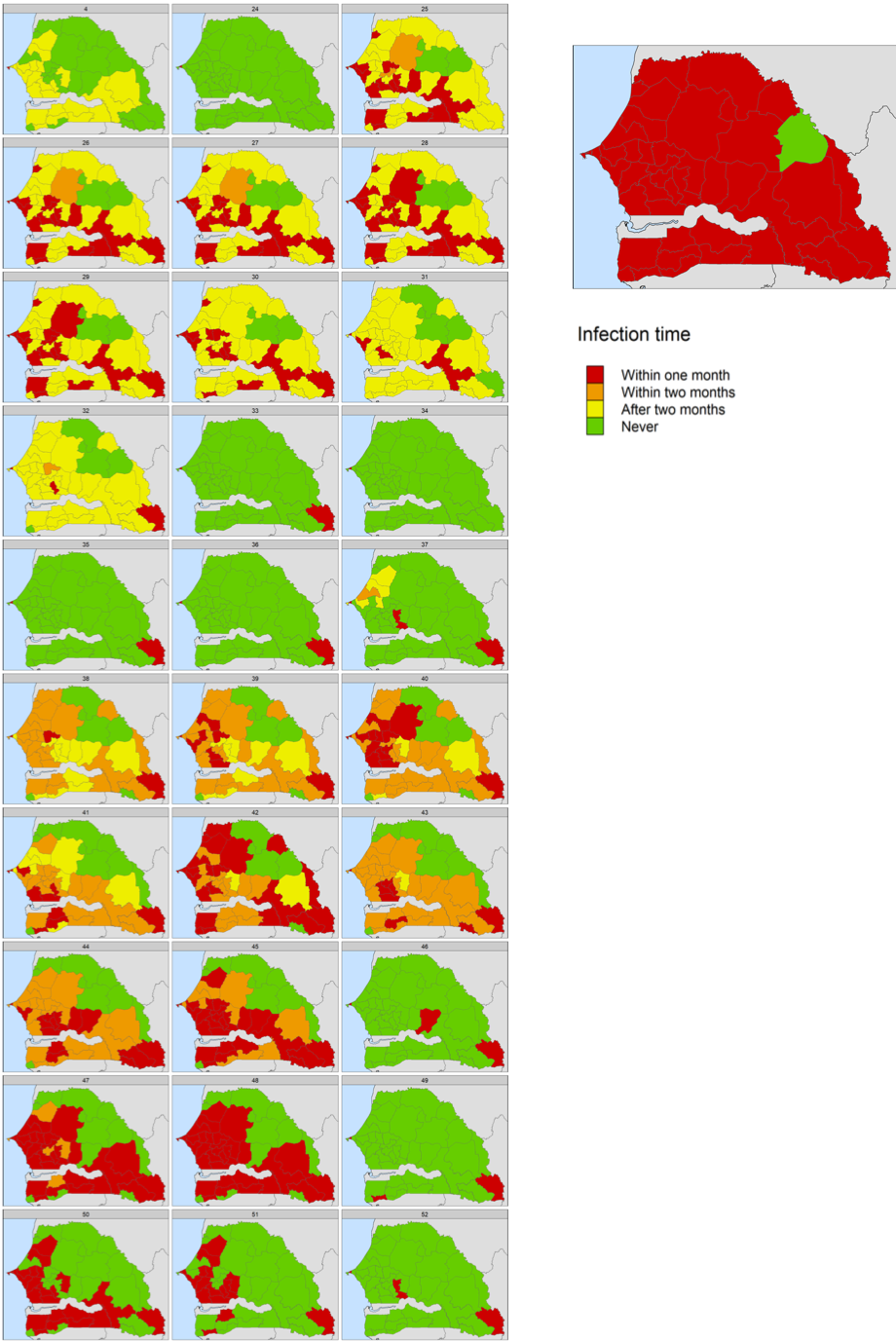 |
| --- |

Figure S5: Geographical representation of infection time, in the case of a disease propagated from Mali through the livestock network. Temporal network on the left, static network on the right. For the static network, the colors are based on the links in the path: up to 5 in red, between 5 and 9 in orange, more than 9 in yellow. Nodes that have never been reached are in green.

| 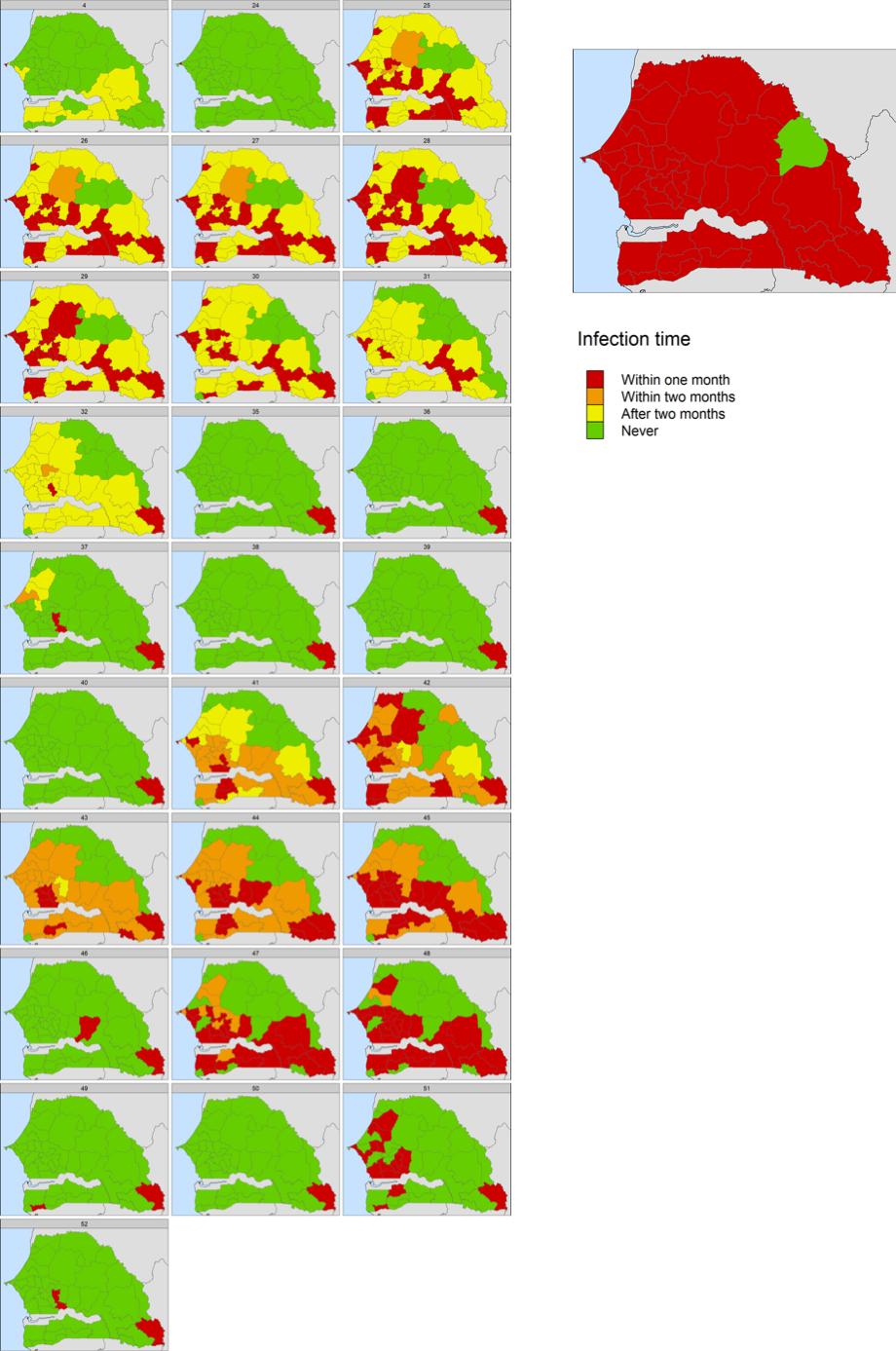 |
| --- |

Figure S6: Geographical representation of infection time, in the case of a disease propagated from Mali through the small ruminant network. Temporal network on the left, static network on the right. For the static network, the colors are based on the links in the path: up to 5 in red, between 5 and 9 in orange, more than 9 in yellow. Nodes that have never been touched are colored green.

| 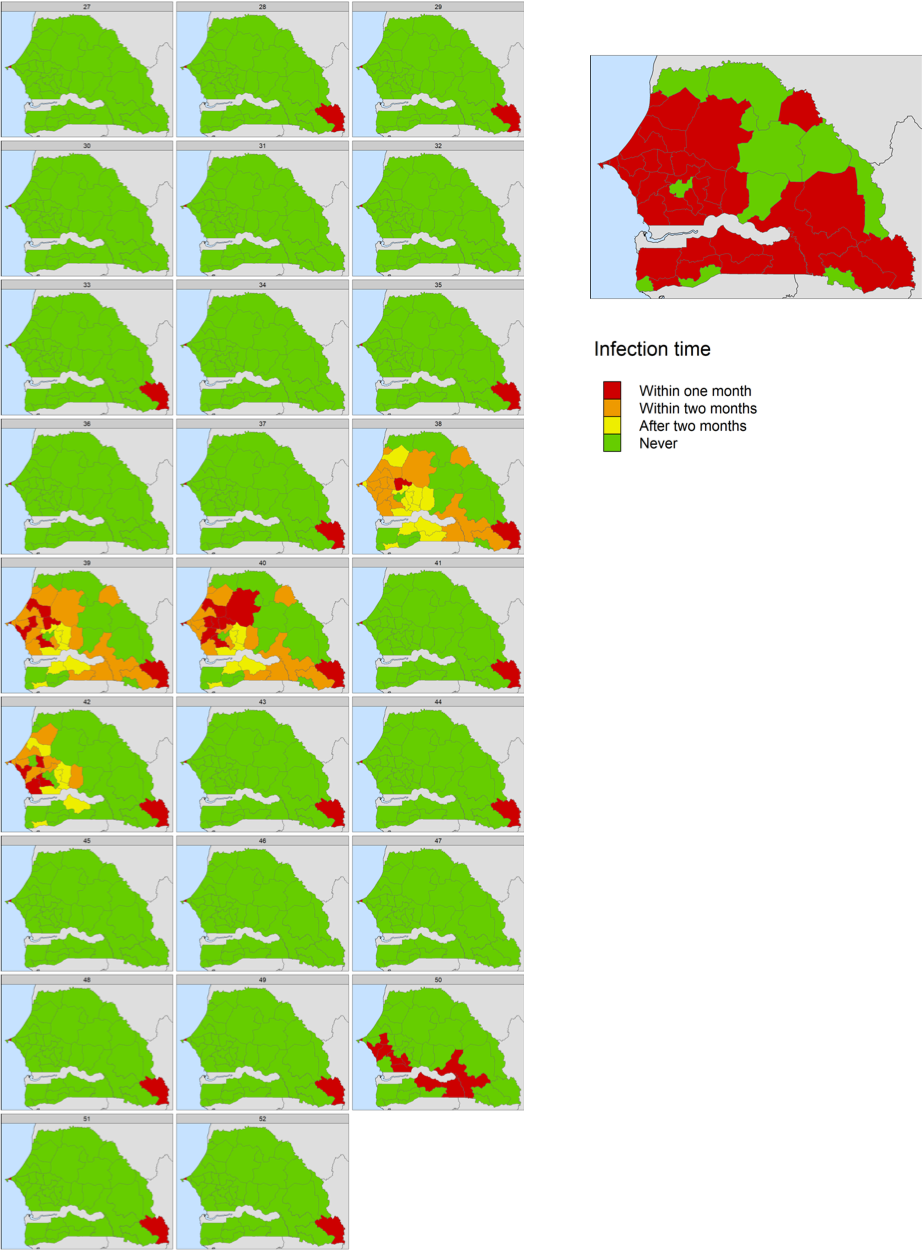 |
| --- |

Figure S7: Geographical representation of infection time in the case of a disease propagated from Mali through the cattle network. Temporal network on the left, static network on the right. For the static network, the colors are based on the links in the path: up to 5 in red, between 5 and 9 in orange, more than 9 in yellow. Nodes that have never been touched are colored green.

| 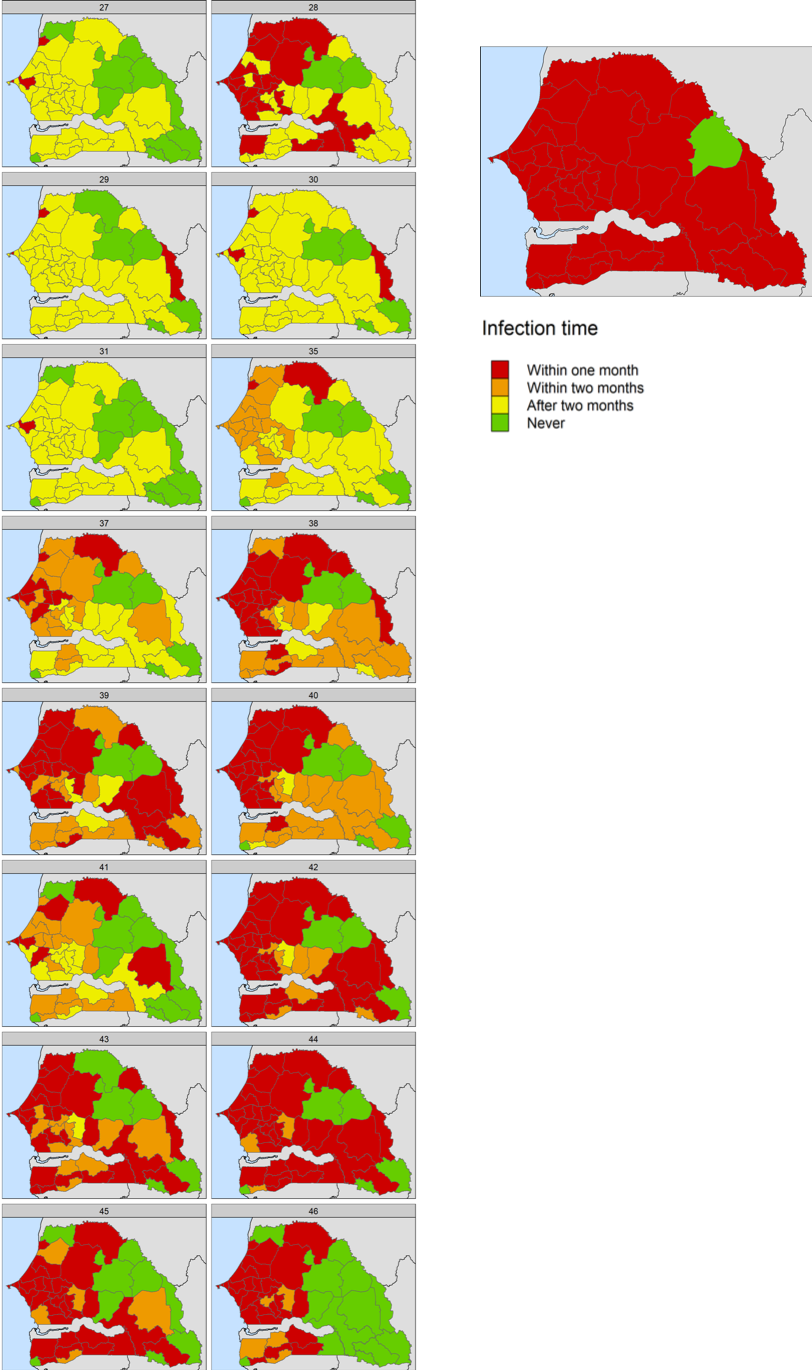 |
| --- |

Figure S8: Geographical representation of infection time in the case of a disease propagated from Mauritania through the livestock network. Temporal network on the left, static network on the right. For the static network, the colors are based on the links in the path: up to 5 in red, between 5 and 9 in orange, more than 9 in yellow. Nodes that have never been touched are colored green.

| 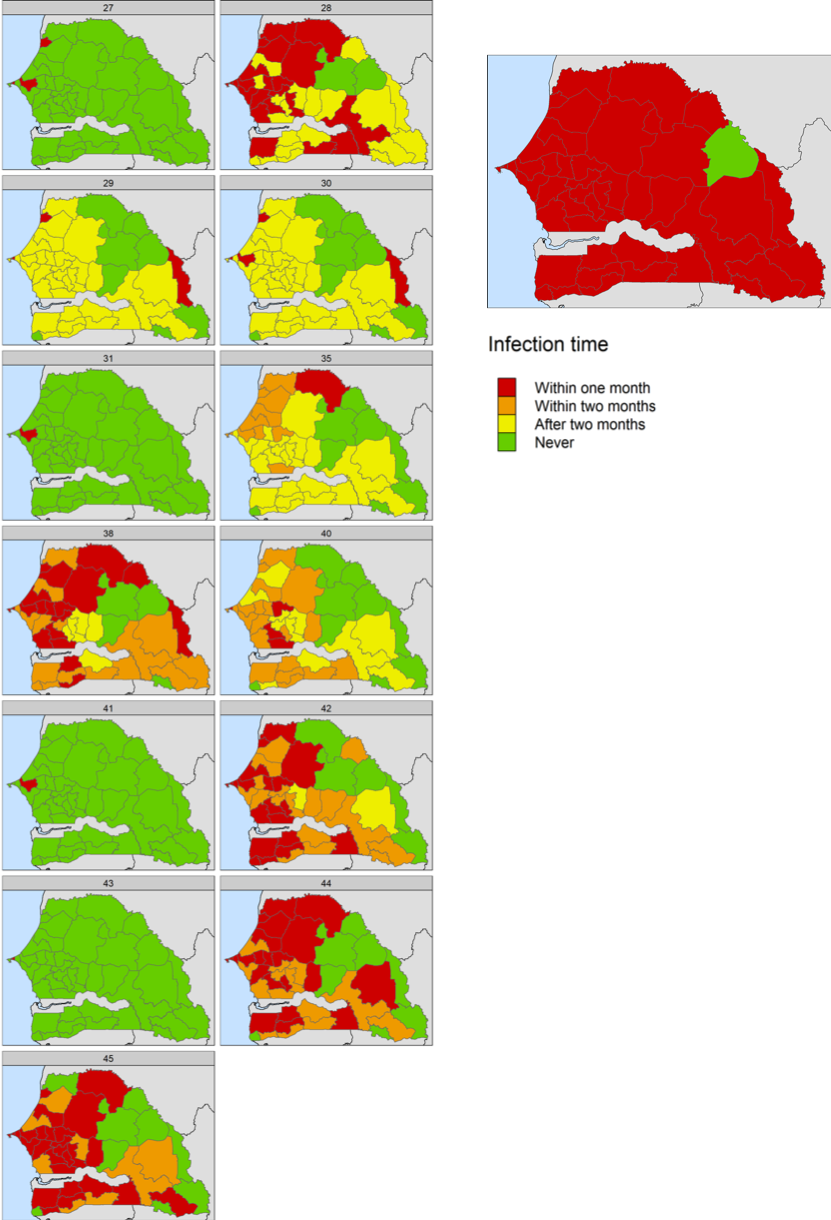 |
| --- |

Figure S9: Geographical representation of infection time in the case of a disease propagated from Mauritania through the small ruminant network. Temporal network on the left, static network on the right. For the static network, the colors are based on the links in the path: up to 5 in red, between 5 and 9 in orange, more than 9 in yellow. Nodes that have never been touched are colored green.

| 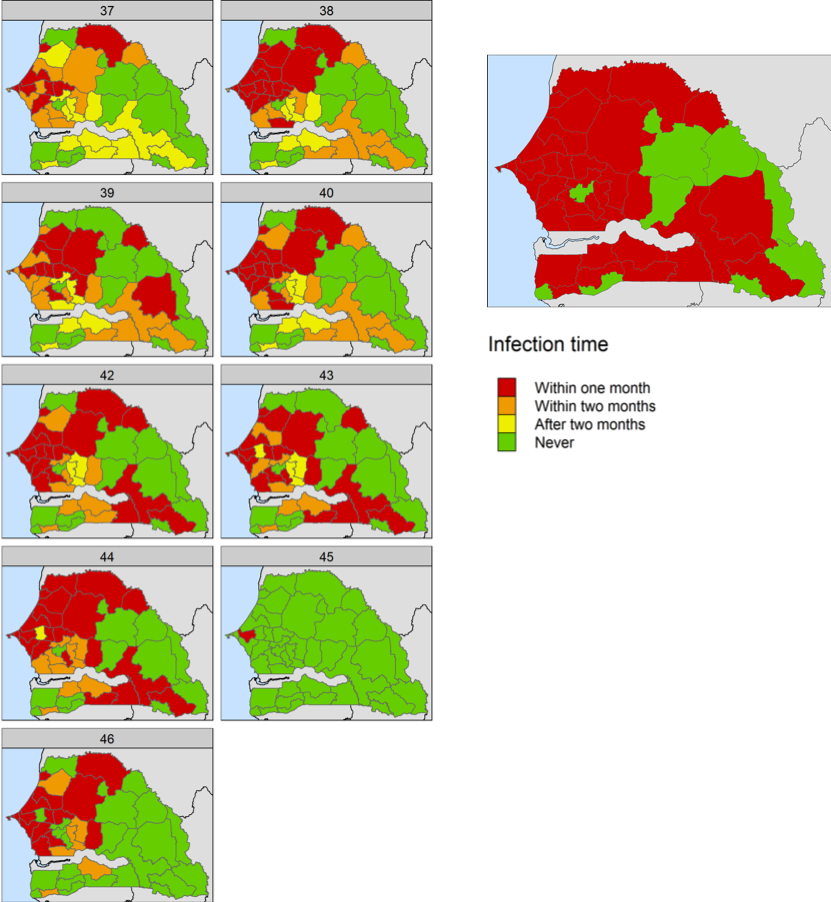 |
| --- |

Figure S10: Geographical representation of infection time in the case of a disease propagated from Mauritania through the cattle network. Temporal network on the left, static network on the right. For the static network, the colors are based on the links in the path: up to 5 in red, between 5 and 9 in orange, more than 9 in yellow. Nodes that have never been touched are colored green.
